# Fault Dynamics of the 1999 Chi-Chi earthquake: clues from nanometric geochemical analysis of fault gouges

**DOI:** 10.1038/s41598-019-42028-w

**Published:** 2019-04-05

**Authors:** Wen-Hsien Li, Chi-Hung Lee, Ma-Hsuan Ma, Ping Jung Huang, Sheng Yun Wu

**Affiliations:** 1 Department of Physics, National Central University, Jhongli, 32001 Taiwan; 2 Department of Physics, National Dong Hwa University, Hualien, 97401 Taiwan

## Abstract

The 1999 Chi-Chi, Taiwan earthquake (Mw 7.6) produced surface ruptures for about 90 km along the north-south trending Chelungpu fault, with surface displacements of up to 12 m. Based on the combination of nanoscopic investigation and geochemistry analysis of core samples from a 450 m long inclined borehole drilled through the slip zone, we suggest the dynamical processes that likely occurred in the northern portion of the Chelungpu fault during the faulting. Our analysis revealed that the frictional heating could have reached 1200 °C, which would cause most of the siderite in the fault gouge to evaporate, resulting in a large amount of nano-size siderite grains with a mean diameter 20 nm. These nano grains could have acted as a mechanical lubricant to reduce the dynamic frictional resistance during sliding, giving rise to the large but smooth type of slipping seen in the north.

## Introduction

The shaking and displacement of the ground seen during earthquakes are the result of a sudden release of energy in the earth’s lithosphere that creates seismic waves which propagate through the lithosphere. During the seismic activity associated with the movement of faults, the grains experience a large shear stress and a tremendous amount of frictional heat is generated that heats the slipping fault to high temperatures. In a large scale earthquake, the temperatures of the slipping zones can reach so high as to cause some of the compounds within the slipping zone to be partially or completely decompose. The changes in the chemical compositions resulting from fault slipping are linked directly to the temperature and shear stress in the faults during slipping^1–3^. Here, we report on the results of studies made on the fault dynamics of the 1999 Chi-Chi earthquake (Mw 7.6) through identifications of the changes in the grain-morphology and chemical composition resulting from fault movement.

Taiwan is located at the collision boundary where the Philippine Sea Plate moves toward the Eurosian Plate in a 310**°** direction at a rate of 70–80 mm/yr. The Chelungpu fault, located on the west coast of central Taiwan, is a reverse fault with a left lateral component that dips moderately to the east. The northern 50 km segment of the Chelungpu fault runs parallel, in the map-view, to the stratigraphic boundary of the hanging-wall of the Pliocene Chinshui Shale and to the bedding immediately adjacent to the base of the Shale. This segment has a ramp-flat geometry, similar to the classic examples of thrust belts, such as in the Appalachians and the Canadian Rockies, and displays fault-bend folding of the thrust at depth, as it steps up from the detachment in the Chinshui Shale.

Out of the thousands of earthquake that occurred in Taiwan in the 20^th^ century, the 1999 Chi-Chi earthquake (Mw 7.6) along the Chelungpu fault was the largest in magnitude. It appears that the fault movement along the northern segment was marked by a high velocity (3–5 m/s) and large displacement (over 10 m); whereas a typical inland earthquake, with moderate velocities on the order of 1 m/s and displacements of several meters, was recorded in the southern segment near the epicenter^4–9^. The most striking feature of the coseismic displacement field of the hanging-wall is the areas of large surface displacement lying above the ramp of the thrust and at the northern termination point. Relatively smaller but significant (1–3 m) displacements are recorded at the detachment point. The horizontal component of the surface displacement increases from the south (less than 1 m) to the north (~3 m), and reaching about 10 m at the northern end near the Shihgang area. The vertical uplift ranges from tens of centimeters to 3.5 m at the southern end, and gradually increases northward to 4 m at Takeng, with a maximum of 7 m near Shihgang^10,11^.

## Materials and Methods

**Figure 1:**
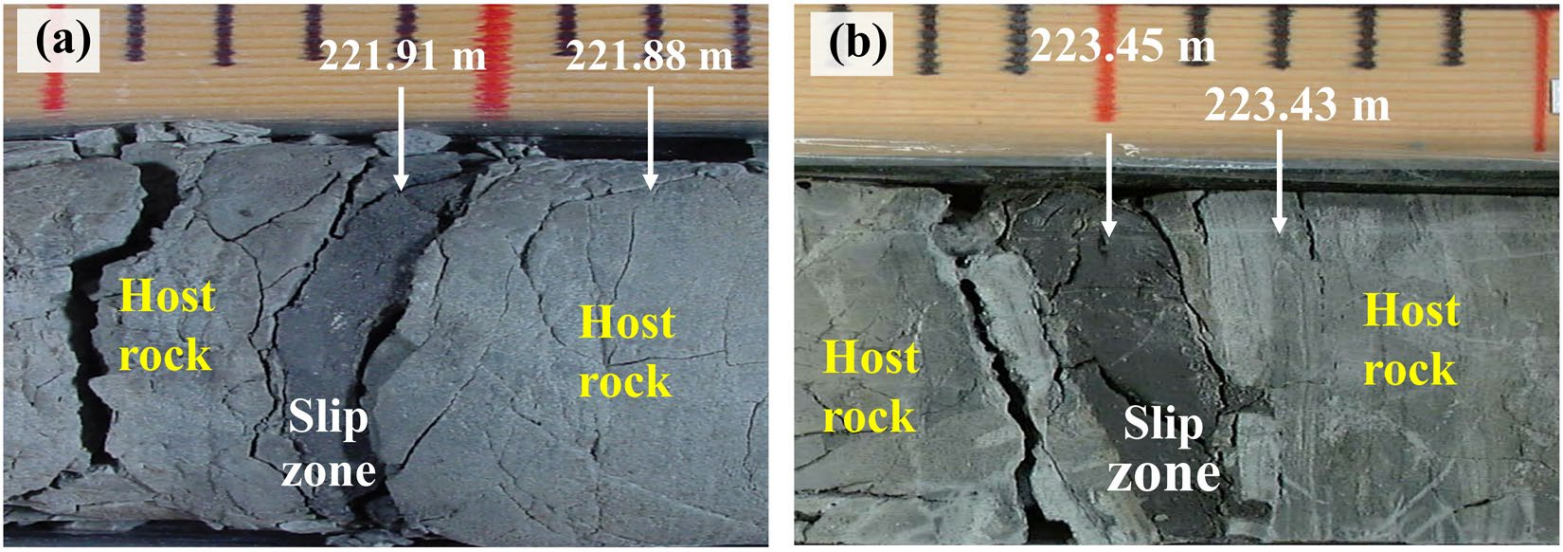
Optical images of the fault zones that slipped during the 1999 Chi-Chi earthquake at depths of (**a**) 221.91 m and (**b**) 223.45 m, recovered in the Fengyuan borehole. Highly localized narrow zones ~2 cm thick of slipped fault gouges are visible. The nearby ambient host rocks are relatively undeformed.

Two shallow boreholes, one at the northern end (Fengyuan well) and the other at the southern end (Nantou well) were drilled to penetrate through the fault zones and the core materials were recovered. Descriptions of the cores from both wells have already been documented^12,13^. Among the many fault zones observable in the Fengyuan borehole, two major ones are identified as possibly having slipped during the 1999 Chi-Chi earthquake. One of these is a highly localized narrow zone (~2 cm thick) of fault gouge, comprised mainly of clay-sized particles, found at a depth of 221.91 m (Fig. 1a) as well as at a depth of 223.45 m (Fig. 1b), where the nearby ambient host rocks are relatively undeformed. The second one shows a composite architecture comprised of both fault gouge and damage zones (fault breccia) that developed at a depth of nearly 330 m. The justification for assuming that these fault zones were activated during the 1999 Chi-Chi earthquake has already been discussed^12–14^.

Taking the advantage of atomic force microscopy (AFM) is capable of imaging three-dimensional shape of a sample surface with a resolution on the order of fractions of a nanometer. The grain-morphology of the recovered cores was examined by an AFM. The AFM images were taken using a Digital Instruments Nanoscope III operated in the Tapping Mode, where a noncontact technique, with the cantilever tip vibrating at a large amplitude to avoid trapping, was used to profile sample surface. The scan speed was three lines per second, with scans covering a wide range (up to 15 × 15 μm^2^) to obtain conceivable descriptions of the surface. The geochemical composition of the cores recovered near the slip zones was studied by powder x-ray diffraction measurement, performed on a Bruker D8 ADVANCE diffractometer, employing the standard reflection geometry. The relative fraction of each composition in the gauge was obtained by quantitatively refining the x-ray patterns, employing the General Structure Analysis System program, following the Rietveld refinement method^15^.

## Results and Discussion

The behavior of the 1999 Chi-Chi earthquake has been documented in many studies^16–21^. In this study, we focus on the important question of what dynamic processes might have occurred within the fault zone during the slipping that would give rise to the heterogeneous large slipping at the northern end of the Chi-Chi earthquake with a high slipping velocity and large ground displacement. It is certain that the quite different fault motions that took place along the northern and southern segments during the Chi-Chi earthquake are directly related to the heterogeneity of the fault rocks. Several key factors played a contributing role. Firstly, the elevated Chelungpu ramps and flats at the northern termination point might have resulted in a reduction of the local overburden and frictional stress, as the areas of large displacements (3–10 m) lie entirely above the elevated Wufeng frontal ramp and the Fengyuan transverse ramp. Secondly, the appearance of thin layers of very soft fault clay in the borehole cores recovered from the borehole at the north suggest^22^ that elastohydrodynamic lubrication^23^ played a role. An incipient pseudotachylyte was found in samples from the south. However, smectite dehydrates at ~250 °C and pore water vaporizes at ~180 °C, indicating that elastohydrodynamic lubrication would only work for a limited depth range and at the beginning of the faulting process, when the frictional heating was not yet severe. There are apparently key factors other than pore water that played a role in elastohydrodynamic lubrication at the later stage of the faulting process. Thirdly, the development of black clayey fault gouge zones in the north (Fig. 1a,b) also suggest that thermal pressurization^24^ might have taken place and that frictional heating produced a significant increase in the slip zone temperature during faulting.

**Figure 2:**
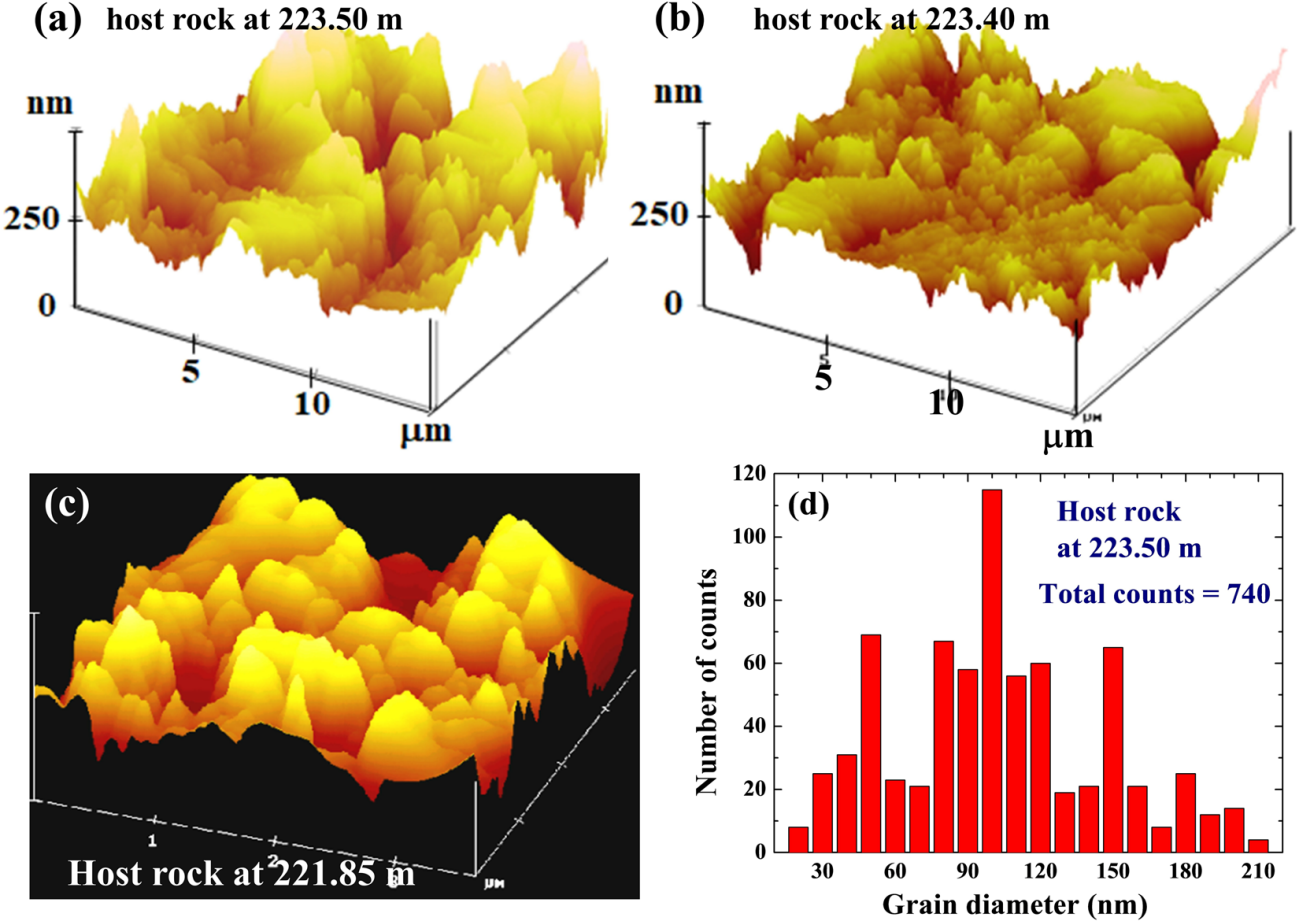
Representative AFM images of the core samples, taken on the host rock from depths of (**a**) 223.50 m, (**b**) 223.40 m, and (**c**) 221.85 m. Enlarged vertical scales are used for clarity of presentation of the stacking heights. Micrometer-sized grains with a sub-µm surface roughness are seen in the images. (**d**) Size distribution of the surface roughness of the host rock at 223.50 m, revealing a surface roughness covering a wide range of up to ~200 nm.

**Figure 3:**
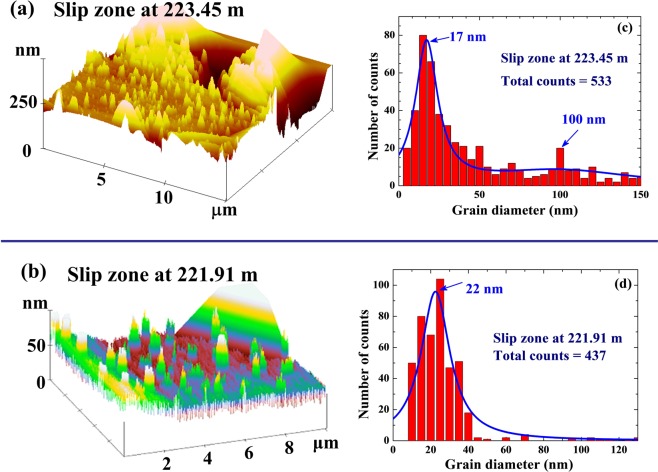
Representative AFM images of the core samples, taken on the slip zone at depths of (**a**) 223.45 m and (**b**) 221.91 m. Enlarged vertical scales are used for clarity of presentation of the stacking heights. A large amount of nano-sized grains and vacant spaces are seen in the images taken within the slip zone. The presence of vacant spaces in the slip zone indicates that some substance has escaped through the slipping process. (**c**) Size distribution of the nano-sized grains at the depth of 223.45 m, revealing a mean grain diameter of 17 nm together with a small fraction of grains with a diameter of around 100 nm. The solid line is a guide to the eyes only. (**d**) Size distribution of the nano-sized grains at the depth of 221.91 m, revealing a mean grain diameter of 22 nm. The solid line is a guide to the eyes only.

Portions of the representative images of the host rock and within the slip zone are shown in Figs 2 and 3, respectively. Grains in µm-sized with a sub-µm surface roughness can be seen in the images of the host rocks obtained at the depth of 223.50 m (Fig. 2a), 223.40 m (Fig. 2b), and 221.85 m (Fig. 2c), revealing a mean surface roughness of around 100 nm but over a wide range of up to 200 nm (Fig. 2d). The images taken within the slip zones are very different. They reveal numerous isolated nm-sized grains but few larger grains (Fig. 3a,b). In addition, the surfaces of the µm grains in the slip zone are much smoother than those in the host rock. Size distribution counts reveal a mean diameter of ~20 nm for both the slip zones at 223.45 nm (Fig. 3b) and at 221.91 m (Fig. 3d). Although many nm-sized grains are revealed in the slip zone, the total volume, hence the total mass, of the nm-sized grains is much smaller (less than 1% when is estimated from the AFM images) than the near-by sub-μm-sized grains. Moreover, vacant spaces can be seen in the slip zone than in the host rock, showing that some substance in the slip zone had vanished, which is consistent with the finding that the mass density of the slip zone is 8% lower. Friction measured by rotary-shear at a slip-rate of 2.12 m/s under a slip stress of 1.18 MPa reveal a dramatic decrease in the friction coefficient of the gouge from 0.9 for the host rock to 0.25 in the slip zone. Nano-sized grains are not normally found in the sediments. These isolated nano-sized grains found in the slip zone were likely to be a result of the slip movement during faulting.

**Figure 4:**
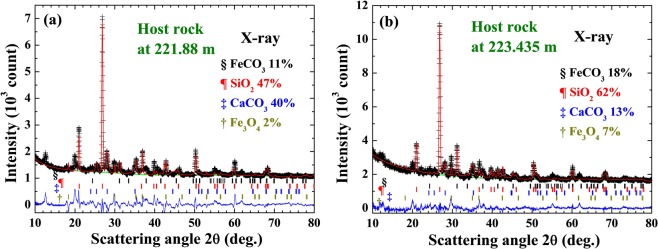
Observed (crosses) and fitted (solid lines) x-ray diffraction patterns collected on the host rocks at depths of (**a**) 221.88 m and (**b**) 223.435 m. The differences between the observed and fitted lines are plotted at the bottoms. Four structural phases belonging to hexagonal quartz (¶), trigonal siderite (§), trigonal calcite (‡), and tetragonal magnetite (†) can be clearly identified. The short vertical lines shown below the pattern mark the calculated positions of the Bragg reflections for each chemical phase.

Four main structural phases, belonging to quartz (hexagonal SiO_2_), siderite (R-centered trigonal FeCO_3_), calcite (trigonal CaCO_3_), and magnetite (tetragonal Fe_3_O_4_) can be clearly identified in the diffraction patterns of the host rock at 221.88 m (Fig. 4a) and at 223.435 m (Fig. 4b). The solid curves in Fig. 4 indicate the calculated patterns with the differences between the observed and calculated patterns are plotted at the bottom. They very good agreement shows that the main compositions of the gauge have been identified. These host rocks are composed mostly of quartz (40% at 221.88 m and 62% at 223.435 m) and are rich in siderite (11% at 221.88 m and 18% at 223.435 m).

**Figure 5:**
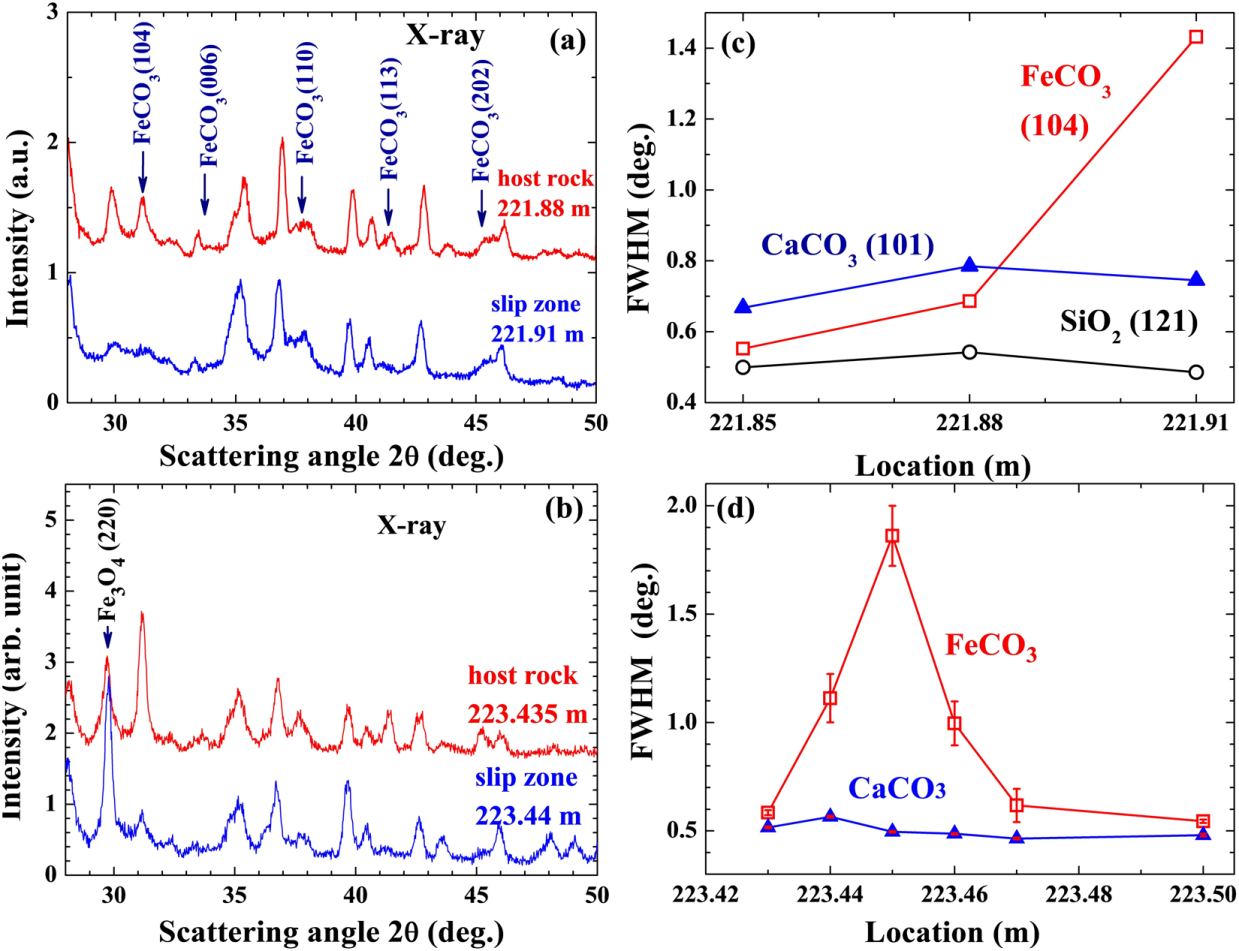
(**a**) Direct comparison between the x-ray diffraction patterns taken on the slip zone at a depth of 221.91 m and the nearby host rock at 221.88 m. The arrows indicate the Bragg positions of siderite. (**b**) Direct comparison between the x-ray diffraction patterns taken on the slip zone at a depth of 223.44 m and the nearby host rock at 223.435 m. The arrow indicate the (220) Bragg position of magnetite. (**c**) Widths of the diffraction peaks of quartz (open circles), siderite (filled triangles), and calcite (open squares) observed at the locations nearby the slip zone at the depth of 221.91 m. (**d**) Widths of the diffraction peaks of siderite (filled triangles) and calcite (open squares) observed at the locations nearby the slip zone at the depth of 223.45 m.

**Figure 6:**
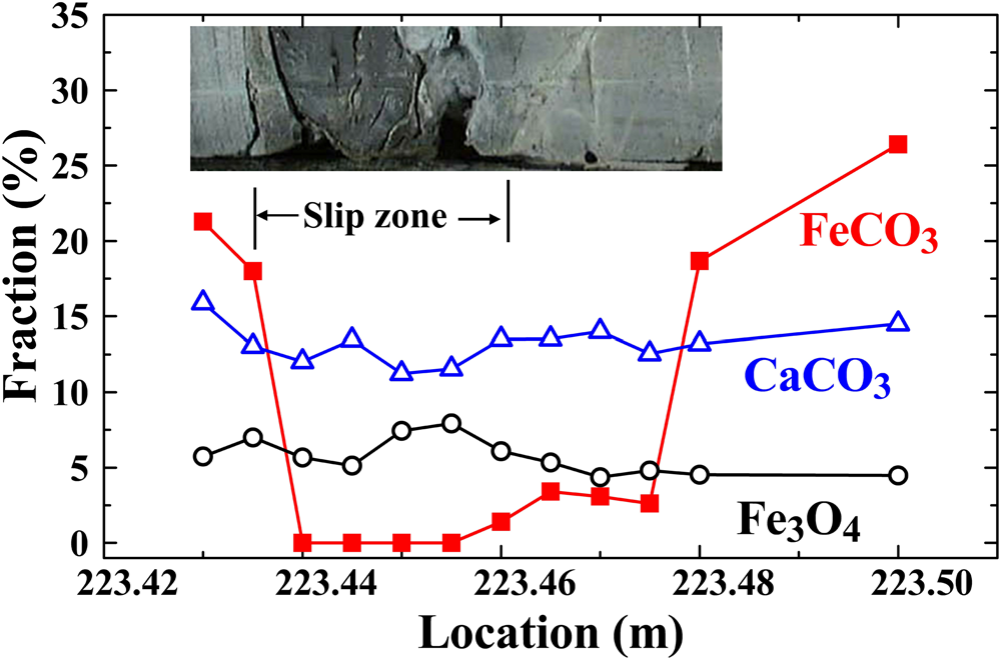
Plot of the refined compositions of siderite, calcite, and magnetite obtained at various locations near the slip zone of the gouge. A photo of the actual specimen from which the measurements were made is shown at the top. The data reveal a dramatic reduction of the amount of siderite in the slip zone, and a relatively small but noticeable reduction of calcite as well.

Diffraction patterns taken within the slip zone and from the nearby host rock reveal the same structural phases. However, the diffraction peaks for the siderite taken in the slip zone are significantly less intense and much broader (Fig. 5a,b). Siderite is still visible but has not completely varnished in the slip zone. The peak width of the siderite in the slip zone is four times broader that of the host rock nearby; whereas the peak widths of quartz and calcite remain essentially unaltered (Fig. 5c,d). This shows that the slip zone contains less siderite and that the siderite grains are nano-sized, so that the finite size effect, which is known to produce such broad diffraction peaks, is revealed in the diffraction profiles. By fitting the pattern corresponding to the siderite in the slip zone to the diffraction profiles of finite sized particles^20^, we obtained a mean particle diameter of 20 nm for the siderite grains in the slip zone. This mean diameter obtained from the x-ray diffraction profiles agrees well with the mean diameter of the nano grains found in the AFM images shown in Fig. 3. The nano grains seen in the AFM images are hence identified to be siderite. Note that the presence of nano-sized grains and empty spaces might act as a mechanical lubricant encouraging fault sliding, due to the reduction in the interlocking of surface asperities^21^, which would greatly reduce the dynamical friction and the generated frictional heat. The refined fractions of the siderite, calcite, and magnetite obtained at locations around a slip zone are shown in Fig. 6. A photo of the actual specimen from which the x-ray diffraction measurements were made is also shown. A dramatic reduction in the amount of FeCO_3_ in the slip zone is clearly revealed, going from 21% in the host rock to almost completely disappearing in the slip zone, while a less dramatic but still noticeable amount of reduction is also found in CaCO_3_. These observations suggest that the increase in the temperature in the slip zone during faulting, likely due to frictional heating, caused the FeCO_3_ to evaporate, with many nm-grains left over.

**Figure 7:**
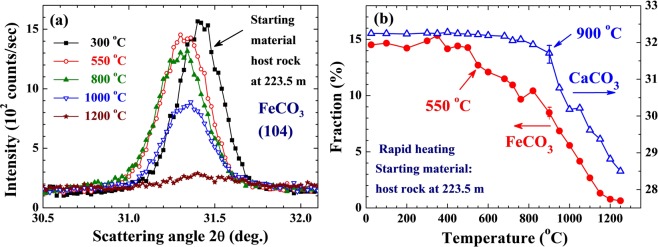
(**a**) Changes of the representative (104) diffraction intensity of the siderite in the host rock at the depth of 223.5 m after being heated in a Rapid Thermal Annealing Furnace with a heating cycle of 25 sec. (**b**) Variations of the composition of siderite (filled circles) and calcite (open triangles) in the host rock at the depth of 223.5 m after the Rapid Thermal Annealing treatment. It can be seen that the siderite begins to evaporate at ~550 °C with a steady reduction to the amount as that found in the slip zone at ~1200 °C.

We can further simulate this dissociation process by measuring the changes in the compositions of the host rock by heating them in a furnace^25–28^. This experiment was performed on a host rock from 223.5 m located beside the slip zone, using a Rapid Thermal Annealing Furnace with a heating cycle of 25 sec for each temperature change. The results are displayed in Fig. 7. It can be seen that the FeCO_3_ in the host rock begins to dissociate at ~550 °C (Fig. 7a), and that significant dissociation continues to occur until 1200 °C, at which point the FeCO_3_ is reduced to essentially the same amount as that found in the slip zone (filled circles in Fig. 7b). As expected, a less significant but noticeable decrease in the amount of CaCO_3_ is also seen above ~900 °C (open triangles in Fig. 7b). Note that the furnace-heating experiment was conducted at atmospheric pressure, whereas the sliding during the earthquake occurred at a vertical depth of 160 m corresponding to a lithostatic load of about 40 atm. However, the decarbonation curve for FeCO_3_ is known^22^ to shift by 100 °C at 10^4^ atm. There may not be a significant difference in the dissociation temperature of FeCO_3_ between at 1 atm and at 40 atm. We estimate that the temperature in the slip zone could have reached 1200 °C during the faulting process, which would have caused most of the FeCO_3_ and some of the CaCO_3_ to evaporate. The energy consumed by evaporation of FeCO_3_ and CaCO_3_ is estimated to be ~4×10^9^ erg/cm^3^. We further note a noticeable increase in the amount of Fe_3_O_4_ (about twice the normal amount) in the slip zone (Fig. 6), which agrees with the magnetic susceptibility of the gouge in slip zone being 2.3 times higher than that of the host rock. Fe_3_O_4_ is known to be a resultant compound of FeCO_3_ oxidation^23^. The increased amount of Fe_3_O_4_ in the slip zone is apparently a by-product of the dissociation of FeCO_3_. However, the amount of increase in Fe_3_O_4_ can account for only 12% of the FeCO_3_ that has disappeared. This, we believe, is because there was not enough O_2_ in the slip zone, so most of the FeCO_3_ evaporated and escaped, leaving noticeable vacant spaces in the slip zone, as revealed in the AFM images shown in Fig. 2. A similar behavior was also observed in other slip zones at the Fengyuan well.

## Conclusions

We suggest the occurrence of the following dynamical process during the faulting, which led to the large displacement and low frequency of ground motion in the northern segment of the Chelungpu fault during the 1999 Chi-Chi earthquake. The Chinshui Shale near Fengyuan city is rich in siderite (~21%), which has a relatively low evaporation temperature, and is saturated with formation water, which has an evaporation temperature of ~180 °C at a depth of 160 m. Once faulting was initiated, frictional heating raised the temperature of the slip zone. When it reached 180 °C, pore water began to vaporize, generating a huge vapor pressure, which in turn further enhanced further faulting. The temperature of the slip zone continued to rise, reaching 1200 °C, which caused most of the siderite and a portion of the calcite to evaporate, leaving vacant spaces and many nano siderite grains as a result. The presence of vacant spaces reduced the contact area, and the spherical nano-grains acted as ball-bearings. Together they caused a significant reduction in the dynamical friction, resulting in the large fault displacements. Finally, the nano-grains supported sliding which generated much less frictional heat. In the meantime, the evaporation of water and siderite consumed a large amount of energy. Consequently, the energy left over would be much less than what would originally have expected. We believe that this is the main reason why no residual heat was observed in the shallow boreholes penetrating the Fengyuan fault zone.
